# Identifying sex-linked markers in *Litoria aurea*: a novel approach to understanding sex chromosome evolution in an amphibian

**DOI:** 10.1038/s41598-019-52970-4

**Published:** 2019-11-12

**Authors:** Jarrod Sopniewski, Foyez Shams, Benjamin C. Scheele, Ben J. Kefford, Tariq Ezaz

**Affiliations:** 1 Institute for Applied Ecology, University of Canberra, Bruce 2617, Canberra, Australia; 2 Fenner School of Environment and Society, The Australian National University, Canberra, ACT 2601 Australia

**Keywords:** Genetic markers, Evolutionary genetics, Evolutionary biology

## Abstract

Few taxa exhibit the variability of sex-determining modes as amphibians. However, due to the presence of homomorphic sex chromosomes in many species, this phenomenon has been difficult to study. The Australian frog, *Litoria aurea*, has been relatively well studied over the past 20 years due to widespread declines largely attributable to chytrid fungus. However, it has been subject to few molecular studies and its mode of sex determination remained unknown. We applied DArTseq™ to develop sex-linked single nucleotide polymorphisms (SNPs) and restriction fragment presence/absence (PA) markers in 44 phenotypically sexed *L. aurea* individuals from the Molonglo River in NSW, Australia. We conclusively identified a male heterogametic (XX-XY) sex determination mode in this species, identifying 11 perfectly sex-linked SNP and six strongly sex-linked PA markers. We identified a further 47 moderately sex-linked SNP loci, likely serving as evidence indicative of XY recombination. Furthermore, within these 47 loci, a group of nine males were found to have a feminised Y chromosome that significantly differed to all other males. We postulate ancestral sex-reversal as a means for the evolution of this now pseudoautosomal region on the Y chromosome. Our findings present new evidence for the ‘fountain of youth’ hypothesis for the retention of homomorphic sex chromosomes in amphibians and describe a novel approach for the study of sex chromosome evolution in amphibia.

## Introduction

**Figure 1:**
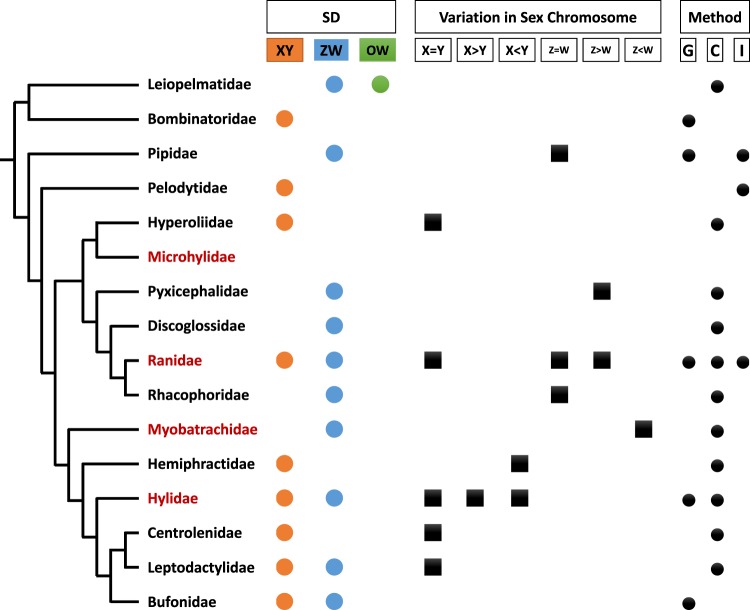
Truncated phylogeny (not according to scale) showing variations of sex determination modes and sex chromosome morphologies among different Anuran families. Australian frog species belong to red coloured families. G = genetic, C = cytological, I = immunologic. Lack of symbols indicate an absence of data. Summarized from Hillis and Green (1990)^56^, Schmid and Steinlein (2001)^57^, Sarre *et al*. (2011)^58^, Miura (2017)^59^ and Ito (2018)^60^. Phylogeny adopted from Jetz and Pyron (2018)^61^.

The biological process of sex-determination amongst vertebrates is diverse and widely studied^1–6^, and few taxa exhibit the variability of sex-determining modes as that of the amphibians^1,5,7,8^. Specifically, within the anurans (frogs and toads), multiple variations of sex chromosome systems have been identified, with both male heterogamety (XX/XY)^9^ and female heterogamety (ZZ/ZW)^6^ being observed. Within anurans both XY and ZW sex chromosome systems have evolved multiple times. In addition, changes in the heterogametic sex have been observed involving the same chromosome pairs (i.e. XY chromosomes become ZW chromosomes) as reported in the Japanese frog *Glandirana rugosa*^10^, thereby suggesting frequent and easy transitions between heterogametic systems (Fig. 1). Despite all amphibian species studied thus far exhibiting genetic sex determination (GSD)^1^, phenotypic sex has been shown to be influenced by both temperature^5,11^ and endocrine disrupting chemical pollutants^7,12^. Adverse temperatures and pollution have resulted in sex reversal being observed in various anuran species, such as *Rana sylvatica*^5^ and *G. rugosa*^13^. Despite the identification of some amphibian sex determining modes^14^, in the context of the unique biosphere of Australia, knowledge of the sex determining mechanisms in amphibians is sparse. To our knowledge, no sex-linked markers have been reported for an anuran species in Australia, nor has sex reversal been noted in any Australian anurans.

The study of sex-determining systems in anurans has been difficult; many amphibians have homomorphic sex chromosomes^15^, thus sex chromosomes cannot be easily identified using conventional karyotyping. A variety of methods have been utilised to genotypically assign sex, such as the examination of inheritance patterns of allozymes^16^, identification of sex-linked microsatellite loci^3^ and more recently genotyping-by-sequencing (GBS) methods such as RAD-seq.^17^ (Fig. 1). DArTseq™, developed by Diversity Arrays Technology Pty Ltd, has been proposed by Lambert *et al*.^9^ as an effective method to identify sex-linked markers in non-model species using single nucleotide polymorphic (SNP) loci, which also generate restriction site specific presence-absence (PA) markers. In comparison to the aforementioned methods, DArTseq™ provides a relatively inexpensive, simple and reliable source of genetic data, which can also be utilised further for additional population analysis^9,18,19^. DArTseq™ combines the genome complexity reduction method DArT, first reported in 2001^20^, with modern next generation sequencing (NGS) platforms. The technology integrates high throughput processing of DNA to generate complexity reduced genome representations using combinations of restriction enzymes selecting the optimal fraction of the genome for sequencing with dedicated analytical processes^19^. This method has been successfully deployed in many plant^18^ and animal^21^ species. DArTseq™ allows for a lower density of high-quality markers, providing reasonable coverage of the genome with a low level of excluded data^18^. The markers generated by DArTseq™ reveal loci assorted to a particular sex, thereby accurately identifying loci tightly linked to the sex-determining region of the sex chromosomes, as such proving to be a useful molecular tool for the deciphering of sex-determining modes in non-model species with cryptic sex chromosomes. Furthermore, through the observation of discordance between genotypic and phenotypic sex, these sex-linked markers allow sex-reversal to be identified in non-model species^9^.

In amphibians, sex reversal has been observed frequently both in the laboratory^22^ and in natural populations^12,23–25^. As previously mentioned, the sex chromosomes of amphibians are largely unique among taxa, both due to the common occurrence of homomorphy as well as transitions between sex-determining modes. The retention of homomorphy between sex chromosomes among amphibians has been shown to be somewhat attributable to occasional recombination between XY sex chromosomes as reported in the hylids *H. arborea, H. intermedia* and *H. molleri*^15^. In these male heterogametic species, XY recombination has been shown to significantly differ from 0, though be less than between the X chromosomes of homogametic females. Furthermore, the ‘fountain of youth’ hypothesis^26^ suggests that sex chromosome recombination is phenotypically moderated, rather than genotypically; in a male heterogametic species, sex chromosome recombination is suppressed by ‘maleness’, rather than differing sex chromosomes^26–28^. As such, sex-reversal has great ramifications for the evolution of the Y chromosome in amphibians; Y recombination may traditionally be suppressed by ‘maleness’, however in an XY female, recombination is instigated, and the Y chromosome is reinvigorated^27^. The identification and analysis of sex-linked genetic markers provides a genotypic tool for the practical sexing of individuals and allows for the detection of sex-reversal in populations and insight into the evolutionary history of the Y chromosome (or W chromosome in a female heterogametic species).

One non-model species from which the identification of sex-linked markers will prove useful to the study of sex chromosome evolution is the Australian frog, *Litoria aurea* (Lesson, 1827 - Anura: Hylidae), or the ‘Green and Golden Bell Frog’. *Litoria aurea*, listed in 2004, is deemed ‘vulnerable’ on the IUCN Red List^29^, and is experiencing ongoing declines^30^ and was thought to have completely disappeared from areas above 250 m in elevation^31^. As such, this species has been relatively well studied over 20 years^32^. The species’ range once extended from the East Gippsland region in Victoria along the eastern coastline to northern New South Wales (NSW), though has since suffered disappearances from over 90% of its range^33^. There is robust evidence that the demise of this species is principally due to the introduction of chytrid fungus^34,35^, although pollution^36^ and the predation of eggs and tadpoles from the introduced fish *Gambusia holbrooki*^32^ may have also contributed. Despite the numerous studies undertaken on this frog, only one has used molecular data, this being a population analysis of several microsatellite loci in the early 2000s^33,37^. Thus far, *L. aurea’s* sex-determining mode is unknown and sex reversal in this species is undocumented. Inclusive of our study population, wild populations that have been studied often exhibit a male bias sex ratio^38,39^. Detection of males is far higher than females due to the loudness of their call, thus this sex bias is likely attributed to sampling bias^40^. However, new sex-linked molecular markers, such as those obtainable from DArTseq™, will allow for exploration as to whether sex reversal is contributing to the reported male bias sex ratio in this species. As a member of the hylids, a family that has already been studied with regard to sex chromosome evolution^15,41^, *L. aurea* acts as a novel model species to study this phenomenon. Furthermore, the markers we have developed not only contribute to the evolutionary understanding of the sex chromosomes of the anuran, they also have potential for future use as a conservation resource, by enabling identification of whether sex-reversal is occurring in wild populations.

In this study, we aimed to address the knowledge gap that exists with respect to the sex-determining modes in an Australian frog, *L. aurea*, as well as identify any sex-linked markers in this taxon. From these markers, we also aimed to postulate the evolutionary history of the sex chromosomes present in this species. Our findings elucidate the previously unknown sex-determining mode, whilst also demonstrating a novel approach to understanding the fascinating interactions between alternate sex chromosomes found in amphibians.

## Results

### Filtration of DArTseq™ data and population analysis

We obtained 40,152 SNP markers and 26,119 DArT silico markers (presence-absence/PA markers) from initial DArT sequencing data. We retained a total of 20,111 SNP markers after filtration following our cut off filtering criteria (call rate = 1, reproducibility = 1). Additionally, we retained 19,121 PA markers after filtering (call rate > 0.90, reproducibility = 1). Initial population analysis utilising these markers suggested the individuals across the three sampling sites to be members of a single, interbreeding population, with little to no population substructure evident between the sites. Pairwise F_ST_ values reported between sampling sites were no higher than 0.026 and the overall population had an F_ST_ value of 0.016, a value generally indicative of little population substructure^42^. Our principal coordinates analysis plot (Supplementary Fig. S1) supports this conclusion, revealing no clustering of individuals by sampling site. Therefore, we considered samples from the three sites to be a single population comprising of 31 males, 13 females and 8 ambiguous individuals whose phenotypic sex could not be conclusively determined in the field. Other studies have shown the degree of a potential marker’s sex-linkage to differ between subpopulations^8,18^; this being a single population, we could assume that any markers correlated with sex at a particular sampling site, though not others, are highly unlikely to be sex-linked.

### Identification and validation of sex-linked markers

Under the parameters outlined in the methods, a total of 11 SNP markers were found to be perfectly linked to maleness, i.e. all males were found to be heterozygous and all females were found to be homozygous for the reference allele at 11 distinct loci (Table 1). In addition, six PA markers were identified as present in over 90% of males and absent in over 90% of females (Table 2). For these selected PA markers, none were found to be present in females or absent in males; however, some results returned ‘null alleles’. No SNP loci were found to assort to females, and no PA markers were noted to be present in over 90% of females and absent in over 90% of males. As such, a male heterogametic sex determination system (XX/XY) has been conclusively determined in *L. aurea*. Furthermore, perfect sex linkage amongst the SNP markers implies that these loci are likely to be tightly linked to the sex determining locus of the sex chromosomes.

**Table 1:** Sequences of both reference (REF) and alternate SNP-containing (ALT) alleles for 11 sex linked SNP loci in *Litoria aurea*, with the position of the polymorphism/s underlined.

Locus	Allele	Sequence	Proportion Homozygous (Reference Allele)		Proportion Heterozygous	
			Male	Female	Male	Female
LiauCT01	REF ALT	TGCAGAAGAAATAGATGAGGTGGAGAGGTGGTAATGAATGTGTGCAGCTATCTTCGTTGTAGCTCATGG TGCAGAAGAAATAGATGAGGTGGAGAGGTGGTAATGCATGTGTGCAGCTATCTTCGTTGTAGCTCATGG	0	1	1	0
LiauCT02	REF ALT	TGCAGAGGAAATGTGTAACAGACCAAATCCGAAATTACATACAGTAGTCACTAACAATTTAAACATGTG TGCAGAGGAAATGTGTAACAGACAAAATCCGAAATTACATACAGTAGTCACTAACAATTTAAACATGTG	0	1	1	0
LiauCT03	REF ALT	TGCAGATATGTACAGAGTGAAGAGGAGGGTGAAAAGGGGAGAAGTCTGAGAGCTGCCAGGATGGATTTG TGCAGGTATGTACAGAGTGAAGAGGAGGGTGGGAAGGGGAGAAGTCTGAGAGCTGCCAGGATGGATTTG	0	1	1	0
LiauCT04	REF ALT	TGCAGATCAGCAGTGCTGTAGAATTGTATGTTGAGCATGGTTTCAAATTATGCAAAGTCCACACCACTG TGCAGATCAACAGTGCTGTAGAATTGTATGTTGAGCATGGTTTCAAATTATGCAAAGTCCACACCACTG	0	1	1	0
LiauCT05	REF ALT	TGCAGCTGGAGCTTCACACCACAAGCAGAGGAGAGCTGTAGCATCAGATAAGCAATAGGAAGTCCAGAG TGCAGCTGGAGCTTTACACCACAAGCAGAGGAGAGCTGTAGCATCAGATAAGCAATAGGAAGTCCAGAG	0	1	1	0
LiauCT06	REF ALT	TGCAGGTGCCTCATTACAGATGCCAACAGGCAGATTTAGGCACTGTTCACACTTGTCTTCTTGCTCTTG TGCAGGTGCCTCATTACAGATGCAAACAGGCAGATTTAGGCACTGTTCACACTTGTCTTCTTGCTCTTG	0	1	1	0
LiauCT07	REF ALT	TGCAGTAGAAGGTGGCTATTGGCATCCTGTTCTTGTCGTGTAACCCAGGGACCTTCAATTGGGCCACTG TGCAGTAGAAGGTGGCTATTGGCTTCCTGTTCTCGTCGTGTAACCCAGGGACCTTCAATTGGGCCACTG	0	1	1	0
LiauCT08	REF ALT	TGCAGTCTGTAAACATGGTTTAAATTTGTTATTGGTAAAAGGAGGCTAAGTATACCCAGGGTAGCTCTA TGCAGTCTGTAAACATGGTTTAAATTTGTTATTGGTAAAAGGAGGCTAAGTATACCCAGGGTAGTTCTA	0	1	1	0
LiauCT09	REF ALT	TGCAGTGCAGTCTGATATACAGTATACTCCCTTATGCAGTTCAAGTGTCTCAAATTGACCATTATCTTT TGCAGTGCAGTCTGATATACAGTATACTCCCTTATGCAGTTCAAGTGTCTCAAAGTGACCATTATCTTT	0	1	1	0
LiauCT10	REF ALT	TGCAGGTAAAACTAGTGTCTCAACAGCCAACTTGTACAGATATTGGTCACTGCATGAGATCGGAAGAGC TGCAGGTAAAACTAGTGTATCAACAGCCAACTTGTACAGATATTGGTCACTGCATGAGATCGGAAGAGC	0	1	1	0
LiauCT11	REF ALT	TGCAGAACATCTTTTCTATGAACTGGTGCCCAGAACTAAACTGCATATTCCAGATGGGGGTCGCATCAA TGCAGAACATCTTTTCTATGAACTGGAGTCCAAAACTAAACTGCATATTCCAGATGGGGGTCGCATCAA	0	1	1	0

All loci perfectly assort to males, indicating a male heterogametic (XX-XY) sex determining system.

**Table 2:** Six sex-linked PA markers.

Locus	Call Rate	Sequence	Male		Female	
			Proportion Present	Proportion Null	Proportion Absent	Proportion Null
LiauCT12	0.980769	TGCAGTAGAAGGTGGCTATTGGCTTCCTGTTCTCGTCGTGTAACCCAGGGACCTTCAATTGGGCCACTG	0.98	0.02	1	0
LiauCT13	0.942308	TGCAGAACTGAGGGCTGATCTACTTTTCGGTAATATTTATGATAAATGCCAAGTCAAAGGGGCAGGTAG	0.90	0.10	1	0
LiauCT14	0.942308	TGCAGTGAGGGATGCCTTACAGGTGCTGTGGCCAGATGCAGAGAAAGAGGATAAGTTACGCTTGTATGT	0.94	0.06	1	0
LiauCT15	0.923077	TGCAGTTGCACAATCTCTTTGCAGAACGTCCTTGCAGAGTTATTTGCAAACCACCCATTTGCAAAGTCT	0.94	0.06	1	0
LiauCT16	0.942308	TGCAGAGCATTGGGTTCACAGTGAAGTCCCCTTTATCTAGGTAGGTGACAAGTCACCTGGAATGCTTAT	0.90	0.10	1	0
LiauCT17	0.942308	TGCAGCTTTATGAAACTCCTCTTACACAGTTTTTCTCATCTTCTTTGAAGCAAAGTCTACACTACCGTT	0.90	0.10	1	0

Call rate refers to the proportion of individuals that returned a result (present or absent). For all loci, where the proportion present or absent for a marker was found to be <1, this was solely due to the presence of a null allele (i.e. a valid result was not returned during the sequencing process). No markers that were present in males were found present in any females, and vice versa.

**Figure 2:**
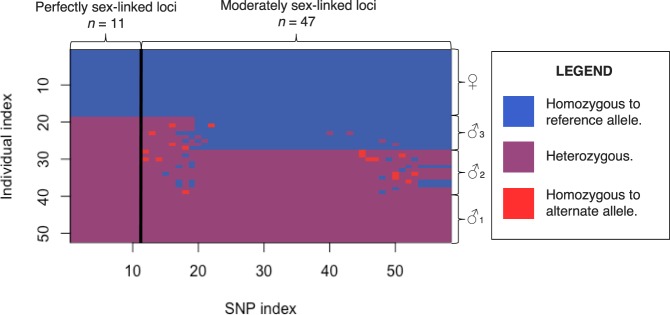
An index of the 11 perfectly sex aligned sequences and the further 47 sex-related sequences created with the ‘glPlot’ function in the R package ‘dartR’^53^. Blue indicates homozygous to the reference allele, purple is indicative of heterozygosity, and red indicates homozygosity to the alternate SNP-containing allele. Sequences 1–11 (left of the figure) show the perfectly sex related sequences, with all females showing blue and all males showing purple. From this point, four clusters emerge: the identical homozygous females, males group 1 (100% heterozygous), males group 2 (high heterozygosity) and males group 3 (low heterozygosity).

For the PA markers, no loci were found to be absent in males, and no loci present in females; for each potentially sex-linked PA marker, some individuals returned a null allele. A null allele appears as a dash in the DArTseq™ file, indicative of a non-zero count recorded, though with too low counts to be considered ‘1’ (present). As such, this often is indicative of heterozygotes. Although it is likely that all males possess the PA marker restriction fragment for each locus, and all females do not, this cannot be confirmed from our data set. As such, though these loci are likely tightly linked to the sex determining region, as with all SNP alleles identified, this cannot be validated without further investigation. Additionally, each of the eight individuals whose sex was not confidently determined in field sampling perfectly conformed to one sex based on each of these selected markers; five perfectly conformed to the female genotype, whereas three aligned perfectly with the male genotype (Fig. 2). This provides validation of our sex-linked markers and overall, the sex ratio of our sample was 65.4% male. This also provides further support to our claim that these markers are indicative of the sex determining region of the sex chromosomes.

**Figure 3:**
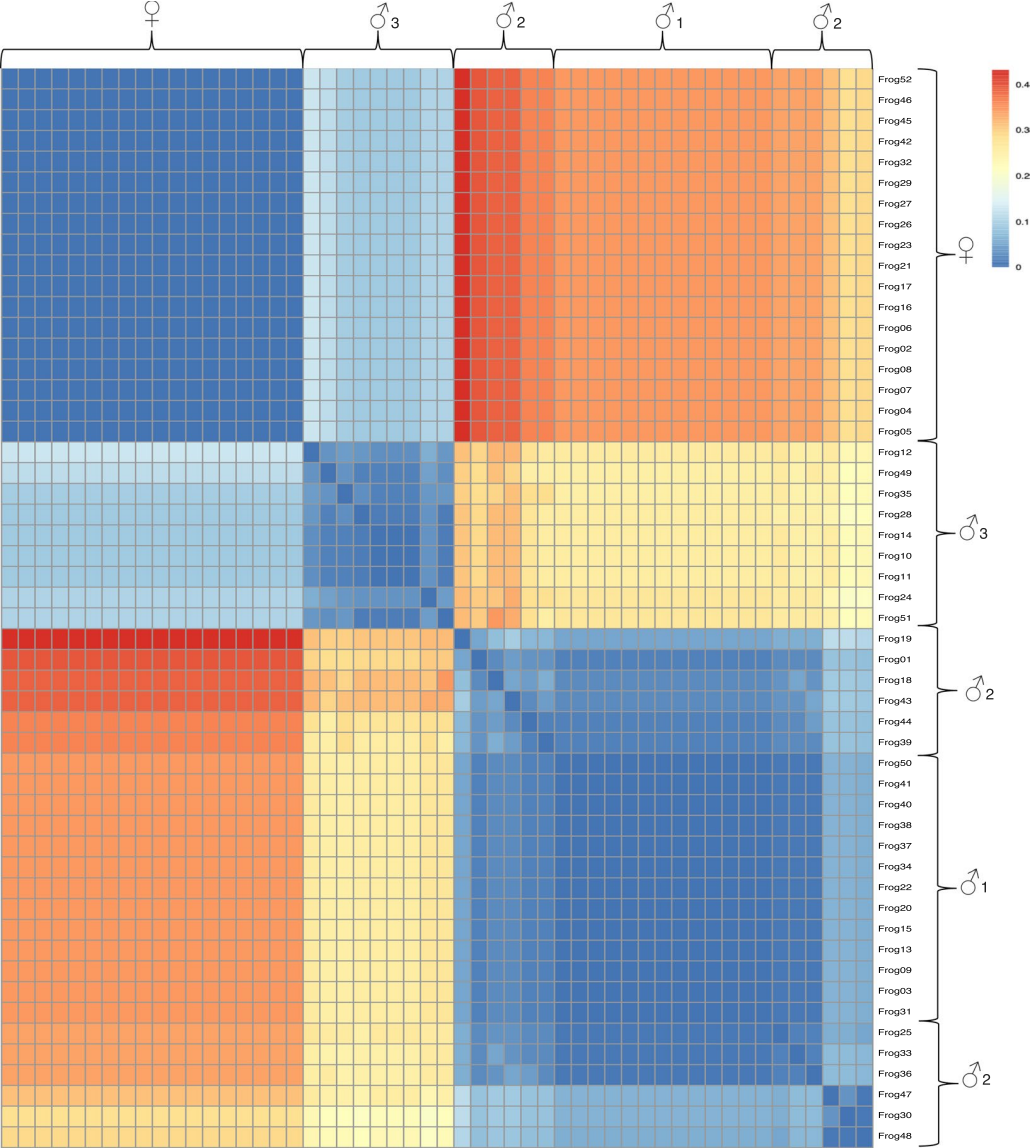
A heat map plotted using the R package ‘pheatmap’^55^ from a genetic distance matrix using the 11 perfectly sex-linked loci and 47 moderately sex-linked loci. The genetic distance matrix was generated using the function ‘stamppNeisD’ from the R package StAMPP^54^. This function calculates Nei’s genetic distance between individuals. Blue indicates identical genotypes, whereas high genetic dissimilarity is displayed in red. Females are genetically identical to each other (homozygous to the reference allele at all 58 loci). Similarly, Males 1, heterozygous at all 58 loci, are identical to each other. Males 2, predominately heterozygous, share high similarity with and are clustered around Males 1, although Males 3 possess a more feminine genotype, hence are more similar to females than their male counterparts.

Furthermore, we identified an additional 47 SNP markers for which each female was homozygous for the reference allele and 13 of the males (herein termed ‘Males 1’) were heterozygous (expressing the genotype for both reference and alternate alleles), when considering the initially non-sex assigned frogs with their genotypic sex. Interestingly, the remaining 23 males that were not perfectly heterozygous assorted into two distinct groups: one group (termed here as ‘Males 2’), comprising of 12 males, were heterozygous for an average of 92.2% (s.d. =5.90%) of the 47 moderately sex-linked loci. The remaining group of individuals, comprising of 9 males (termed here as ‘Males 3’), were heterozygous for an average of 16.3% (s.d. =2.38%) of the 47 moderately sex-linked loci. A two-sample t-Test assuming unequal variances, considering the percent heterozygosity of each individual, shows these two groups to be significantly different to each other (t(15) = 40.38, p = 1.017^−17^, two-tailed). A principal coordinates analysis plot (Supplementary Fig. 2) and a Nei’s genetic distance heat map (Fig. 3) show how the group ‘Males 3’ is more similar to females than their male counterparts when considering the perfectly and moderately sex-linked loci.

### Spurious sex-linkage estimation

Following the model described in Lambert *et al*.^9^, our filtered dataset of 20,111 SNP markers, for a sample of 44 phenotypically sexed individuals, revealed that 1.14 × 10^−9^ markers are likely to be spuriously sex-linked. Similarly, for the PA markers (19,121), 1.08 × 10^−9^ markers would show sex-linkage by chance. As such, it can be stated with confidence that all markers identified are tightly linked with the sex determining locus of this species. As shown in Supplementary Fig. 3, for this number of SNP and PA markers, 14 individuals would be sufficient to be confident that less than one marker would be spuriously sex-linked (adopted from Lambert *et al*.^9^).

## Discussion

Our study successfully identified 11 SNP markers to be perfectly assorted to maleness across all known sex individuals, thus providing decisive evidence that *L. aurea* utilises a male heterogametic sex determining system (XX/XY). In addition, we identified a further six PA markers that are strongly sex-linked; excluding null allele results, each identified marker was present in all males and absent in all females. These results demonstrate the usefulness of the DArTseq™ methodology for identifying sex-linked markers^9^. Furthermore, the remaining non-sex-linked loci obtained from DArTseq™ can be used to conduct a number of population analyses^43–45^.

A further 47 SNP loci were also identified as moderately sex-linked, with all females expressing homozygosity to the reference allele and a total of 13 males recorded as heterozygous across all loci. Of these, eight markers (markers 12–19 inclusive) largely assorted to males (i.e. males were generally heterozygous), as presented in Fig. 2. Here, in the groups ‘Males 2’ and ‘Males 3’, discordance with the heterozygous genotype could reflect potential recombination events between the X and Y sex chromosomes, as has been shown in other hylids^41^. Stöck *et al*. concluded that the evolutionary young sex chromosomes of hylids are the result of occasional recombination between the X and Y chromosomes, rather than turnover events with autosomes^28^. It has been shown on a separate occasion that recombination between the X and Y chromosomes in hylids is significantly greater than 0, though does occur at a rate 10^5^ times slower than recombination between X chromosomes^15^. Partial assortment of these loci to males suggest that these markers were perhaps restricted to the Y chromosome in the past, however have undergone recombination with the X chromosome, hence no longer exhibit perfect male concordance; these potential recombination events are indicated by the alternate coloured rectangles in Fig. 2. This subset of moderately sex-linked markers provide evidence for the occasional recombination between the X and Y sex chromosomes as reported in other hylid species^41^.

When considering the 47 non-perfectly sex-linked markers in their entirety, as previously discussed, the males fall into three distinct groups: ‘Males 1’ (heterozygous for all markers, *n* = 13/34), ‘Males 2’ (mostly heterozygous, *n* = 12/34) and ‘Males 3’ (predominately homozygous to the reference allele, *n* = 9/34). These findings may be indicative of alternate origins of the Y chromosome between these groups. As observed in Fig. 3, ‘Males 1’ appears to be a subset of ‘Males 2’, and we postulate that the minor variations between these two groups may represent further evidence of occasional recombination between the sex chromosomes^41^ as previously discussed; the Y chromosome in these individuals is likely of similar origin. The rate of evolution of sex chromosomes in amphibians is far higher than that observed in other taxa^46^, contributed to by occasional XY recombination^15^. Interestingly, however, our finding of 11 perfectly sex-correlated markers suggest the presence of a distinct male-specific region of the Y chromosome. In order for this formation of distinct sex chromosomal regions to occur and this degree of sex-specific genotypes to be observed, recombination between the X and Y chromosomes in *L. aurea* have significantly slowed^47^. The vast differentiation between the groups ‘Males 3’ and the other males identified is therefore difficult to interpret.

One possible explanation of this phenomenon is the occurrence of a historical sex reversal event in this population that led to the origin of a differentiated Y chromosome, the lineage of males now represented as ‘Males 3’. The ‘fountain of youth’ hypothesis for the retention of homomorphic sex chromosomes in amphibians postulates that recombination between sex chromosomes is suppressed by phenotypic, not genotypic, sex^26,27^. That is, recombination between sex chromosomes is encouraged by ‘femaleness’, and suppressed by ‘maleness’, rather than being influenced by the types of sex chromosomes present^27^. As such, recombination between X and Y chromosomes would be invigorated by sex-reversed XY females; the male phenotype suppresses recombination, thus the female phenotype would allow reinvigoration of the Y chromosome^48^. We postulate that the ‘feminised’ Y chromosome we observe in the ‘Males 3’ group may be resultant from rapid recombination events in an ancestral sex-reversed XY female in a recent sex-reversal event.

Although we posit the occurrence of historic sex-reversal in this population, we did not find evidence of this phenomena currently occurring. During sampling, one individual (Frog31 of Site 2) was tentatively sexed as female in the field, though we were not confident with this morphological sexing, thus for analytical purposes we deemed this individual as ‘unknown sex’. Despite the original tentative female identification, both SNP and PA markers perfectly genotyped this individual as a male. This discordance highlights the greater accuracy of our genetic sexing method over morphological sexing, though also signifies the potential these markers possess for the detection of sex reversed individuals. Pollution in the form of endocrine disruptors, particularly increased levels of estrogens in the environment, has been shown to cause sex reversal in other amphibians^12,24^. Our study population of *L. aurea* resides in an area polluted with metals from a disused mine^49^, and many other populations of *L. aurea* frequent polluted habitats, providing the potential for the occurrence of sex-reversal.

In conclusion, the genotypic markers identified by this study are the first sex-linked molecular markers developed for *L. aurea*, and for an Australian anuran. We were able to identify a notable number of perfectly sex-linked markers given the small portion of the genome represented through DArTseq analysis and the general homomorphy between sex chromosomes often observed in hylids and other amphibians. Furthermore, the 11 perfectly sex-linked markers are indicative of a male specific region of the Y chromosome, present despite major differences observed elsewhere on the chromosome, implying that these markers will likely be sex-linked in other populations. Our results not only elucidated the modes of sex determination in *L. aurea*, though also provide molecular support for both higher rates of recombination between sex chromosomes in amphibians^15,28,41^ and the ‘fountain of youth’ hypothesis for the retention of homomorphic sex chromosomes^26,27^.

## Methods

### Sample collection

In November and December 2016, 52 *Litoria aurea* adults were sampled from the study population on the Molonglo River and associated floodplains in NSW (an exact location cannot be revealed due to a confidentially agreement with the private landowner). At the time of sampling, individual sex (male or females) was determined by the presence or absence of male nuptial pads, male throat discolouration and/or female gravidness; where morphological evidence was inconclusive, the individual was designated ‘unknown sex’. A single digit from each individual was removed at the base of the third phalange and stored in 70% ethanol. Each individual was handled with a new pair of sterile gloves. Field work was conducted under the NSW government scientific licence SL101797 and approved by The Australian National University animal ethics committee (protocol: A2016/25).

### DNA extraction and genotyping-by-sequencing (GBS)

DNA extraction and sequencing were conducted by Diversity Arrays Technology Pty Ltd using DArTseq™, a methodology combining DArT genome complexity reduction methods and next generation sequencing technologies^50^. The methodology used was adapted from similar process utilised by Lambert *et al*.^9^ and Hill *et al*.^21^. The DNA samples were digested using *Pst*I and *Sph*I before ligation reaction were performed using two adaptors: a *PstI* compatible adaptor (consisting of an Illumina flow cell attachment sequence, sequencing primer sequence and a unique barcode sequence), as well as a *SphI* compatible adaptor (consisting of an Illumina flow-cell attachment region). The ligated fragments then underwent 30 rounds of PCR (94 °C for 20 seconds, 58 °C for 30 seconds and 74 °C for 45 seconds), followed by an extension of seven minutes at 72 °C. Following PCR, equimolar amounts of amplification products derived from each individual were bulked and applied to Illumina’s proprietary cBot bridge PCR, which was followed by sequencing on an Illumina Hiseq2000. The single read sequencing was run for 77 cycles.

Proprietary DArTseq™ analytical pipelines were used to process the sequences generated. Initially, the fastq files generated from Hiseq2000 were processed to filter for poor quality sequences. The parameters included the discarding of any results with reproducibility <90% and read depth <3.5 for SNPs and <5 for PA markers, as well as the application of more stringent selection criteria to the barcode region (compared to the remainder of the sequence). Identical sequences were then collapsed into ‘fastqcoll’ files. These were subjected to a secondary pipeline (DArTsoft14), further DArT proprietary pipeline that is differentiated for single nucleotide polymorphism (SNP) and SilicoDArT (presence/absence of restriction fragments in representation). DArTsoft14 utilises a reference-free algorithm whereby each unique sequence from the fastqcoll file is identified and clustered by sequence similarity (three base pair variation is used as the distance threshold) using an optimised clustering algorithm. The resulting SNP and SilicoDArT markers are labelled with a number of metadata parameters derived from the quantity and distribution of each sequence across all samples analysed. The genotyping process utilised by DArTseq™ includes high levels of technical replication, thereby allowing the parameter of ‘reproducibility’ to be calculated, an integral metric.

### Marker filtering and population analysis

The data obtained from DArTseq™ analysis (provided as Supplementary Data) initially contained 40,152 SNP markers and 26,119 presence-absence markers from the SilicoDArT file after the filtering processes undertaken by DArT. The SNP file scored homozygotes to the reference allele as ‘0’, homozygotes to the alternate SNP-containing allele as ‘1’ and heterozygotes as ‘2’. In the SNP file, a result of ‘-‘, known as a null allele, indicates an absence of the fragment containing the SNP in the genomic representation of that individual. SilicoDArT determines genetically ‘dominant’ markers, scored in a binary fashion. Results of “1” indicate presence and “0” indicate absence of a restriction fragment with the marker sequence in the genomic representation of the sample being studied. A null allele result in a SilicoDArT file represents non-zero counts of the restriction fragment, though with too low a calling to confidently score as ‘1’. Call rate refers to the proportion of samples that return a confident result non-null allele results, whereas reproducibility indicates the proportion of technical replicate assay pairs for which the marker score is consistent. As such, we applied additional filtering criteria to the data we received from DArT. For all SNP data, loci with call rate <1 and reproducibility <1 were excluded from analysis. Similarly, for the SilicoDArT file, all loci with a call rate <0.90 and reproducibility <1 were removed from the data set. The call rate threshold was lowered for the SilicoDArT data due to the slightly poorer quality of data received. These stringent filtering methods ensured that only the most reliable sequences were interrogated. These data subsets were used for all subsequent analysis.

Samples were collected from three locations separated by 1–2 kms, and we used the statistical program R^51^ to perform various population analyses. The R package ‘PopGenReport’^52^ was used to calculate the pairwise F_ST_ values between each sampling location using the function ‘pairwise.fstb’. A principal coordinates analysis was also conducted using the functions ‘gl.pcoa’ and ‘gl.pcoa.plot’ of the R package ‘dartR’^53^. The results of these tests were then interrogated to confirm status of our study population as a single population. This was important to ascertain, as other studies have found some sex-linked markers to correlate with geography and differ between subpopulations^8^.

### Selection of sex-linked markers and identification of sex-determining mode

The markers produced by DArTseq™ comprise of two alleles for each SNP locus: a reference allele (the allele most frequently sequenced in the data subset) and an alternative allele (the allele containing a single nucleotide polymorphism with respect to the reference allele). In a male heterogametic (XX-XY) system, the reference allele, sequenced most frequently, would appear on the X chromosome, and the alternate allele would appear on the Y-chromosome. SNP markers that are tightly linked with the sex determining region of the Y chromosome should therefore express 100% heterozygosity in the heterogametic sex (males) and 100% homozygosity to the reference allele in the homogametic sex (females)^12^. For a female heterogametic (ZZ-ZW) system, the opposite would be true. For PA markers, an XX-XY system would be characterised by all males being marked ‘present’ for the marker-containing restriction fragment and all females being marked ‘absent’, and vice versa for a ZZ-ZW system.

We used Microsoft Excel (2015) to test for the sex determining mode used by *L. aurea* and identify and sex-linked markers. Initially, we tested for a male heterogametic system (XX-XY). We manipulated the data such that the columns of results for each individual were arranged in blocks of females, males and unknown sex. For each row, representing each individual for a particular allele, we used the ‘countif’ function to count all heterozygous results (2) for males, divided by 31 (the number of confidently sexed males). For loci where all males are heterozygous, this would give a result of ‘1’; i.e. a proportion of 1 (100%) males are heterozygous. A similar process was used to evaluate the level of homozygosity to the reference allele in females. These two results were then added together, meaning a result of ‘2’ would mean that all males are heterozygous, and all females are homozygous to the reference allele; these would be sex-linked loci. The data was then custom sorted so that each allele that gave a result of ‘2’ appeared at the top of the file, and all alleles were sorted into descending order. The opposite procedure was then conducted to test for a female heterogametic system. A similar approach was used for the SilicoDArT PA subset; however, no loci were found to have 100% concordance with sex. This was largely due to the lower average call rate present in the SilicoDArT file, so we rather selected markers with >90% presence (a result of <0.9) in the heterogametic sex and >90% absence in the homogametic sex and determined these to be sex-linked loci. For all selected PA loci, none returned an ‘absence’ result in males or a ‘presence’ result in females.

### Further analysis

Upon identification of sex-linked loci, we noticed a trend in our SNP data whereby all females continued to be homozygous to the reference allele and most males appeared to be largely heterozygous, though a group of 9 males were homozygous to the reference allele, similarly to females, consistently at the same loci. We identified a further 47 loci for which 13 males were 100% homozygous (Males 1), 12 males were highly heterozygous (>85% heterozygous; Males 2) and the ‘female-like’ group of males expressed low heterozygosity (<20% heterozygosity; Males 3). We deemed these to be moderately sex-linked loci. Using Microsoft Excel, we used the ‘descriptive statistics’ function for Males 2 and Males 3 respectively to determine the mean heterozygosity and standard deviation for each. We then performed a two-sample t-test assuming unequal variances (based off results from the descriptive statistics) to determine if these two groups were significantly different from each other.

We plotted each of these 58 markers against each individual using the ‘glPlot’ function in the R package ‘dartR’^53^. Following this, using the ‘dartR’ functions ‘gl.pcoa’ and ‘gl.pcoa.plot’ respectively, we created a principal coordinates analysis plot to visualise the relatedness between females and the three groups of males identified based on these 58 perfectly and moderately sex-linked loci. Using the same loci, using the R package ‘StAMPP’^54^, we used the function ‘stamppNeisD’ to create a distance matrix based upon the Nei genetic calculated between each individual based upon the 58 isolated loci. We represented this matrix visually through the creation of a heat map, using the function ‘pheatmap’ in the R package ‘pheatmap’^55^.

### Random sex-linkage estimation

Due to the large number of loci provided by DArTseq™, spurious sex association may be possible if analysing a small sample^17^. To ensure the sample size used (*n* = 44 – only confidently sexed samples used) was adequate to minimise the probability of random sex-linked markers being uncovered, the formula presented in Lambert *et al*.^9^ was utilised:

$${P}_{i}={0.5}^{n}$$

Where *P* is the probability that any locus, *i*, is sex-linked by chance, and *n* is the sample size. Following this calculation, the result was multiplied by the number of quality SNP markers analysed to give an estimation of the number of random sex-linked loci produced through SNP analysis, with a similar process being undertaken for the PA markers.

## Supplementary information

Supplementary Figures

Dataset 1

Dataset 2

Dataset 3

Dataset 4
