# Supplementary material for: Identifying sex-linked markers in Litoria aurea: a novel approach to understanding sex chromosome evolution in an amphibian
Source: Sci Rep. 2019 Nov 12;9:16591. doi: 10.1038/s41598-019-52970-4 (PMC6851140; doi:10.1038/s41598-019-52970-4)
Supplement: Supplementary file 1 — Supplementary Figures [file 41598_2019_52970_MOESM1_ESM.docx]

**Identifying sex-linked markers in *Litoria aurea*: a novel approach to understanding sex chromosome evolution in an amphibian**

**Jarrod Sopniewski^1^*, Foyez Shams^1^, Benjamin C. Scheele^2^, Ben J. Kefford^1^, Tariq Ezaz^1^***

^1^Institute for Applied Ecology, University of Canberra, Bruce 2617, Canberra, Australia

^2^Fenner School of Environment and Society, The Australian National University, Canberra ACT 2601, Australia

*Correspondence: Jarrod.Sopniewski@canberra.edu.au; Tariq.Ezaz@canberra.edu.au


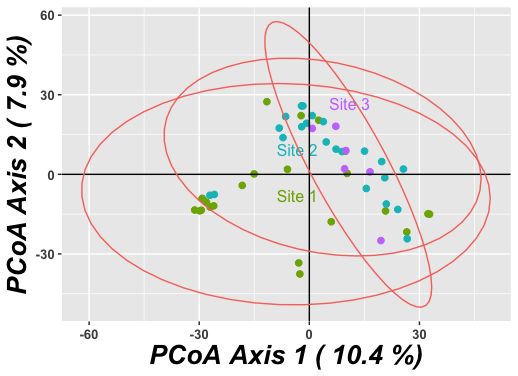


**Supplementary Figure S1.** A principal coordinates analysis plot showing the clustering of each individual sampled with relation to the sampling site based upon all SNP loci provided in the original dataset, using the ‘gl.pcoa.plot’ function in the R package ‘PopGenReport’ ^53^. This supports the conclusions reached from analysis of F_ST_ values between the sampling sites that the exchange of genetic information is not restricted by sampling site in this population, and that each of the individuals we sampled are members of a single, interbreeding population.

**
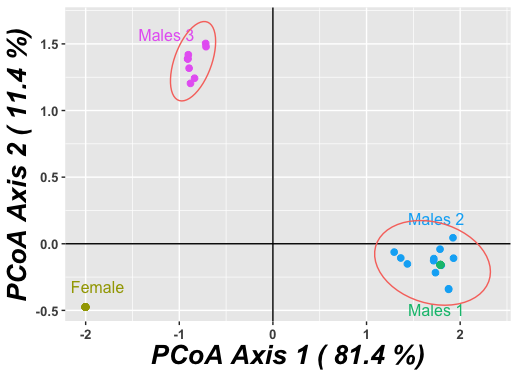
**

**Supplementary Figure S2.** A principal coordinates analysis plot analysing the 11 perfectly sex-linked and 47 moderately sex-linked loci, with individuals designated Female, Males 1, Males 2 or Males 3, as designated in the main text, created with the ‘gl.pcoa.plot’ function in the R package ‘PopGenReport’ ^53^. As can be seen, all females are genetically identical to each other; this is also true of the males in the group ‘Males 1’, as would be expected (each individual has the same genotype, as the markers in these groups are identical). ‘Males 2’ is similar to ‘Males 1’, however the spread of individuals is indicative of recombination events, and not all individuals are identical. The alternate origin of the Y chromosome inherited by males in ‘Males 3’ is evident here; this group is distinctly different to the other males and much more similar to females than their same-sex counterparts.


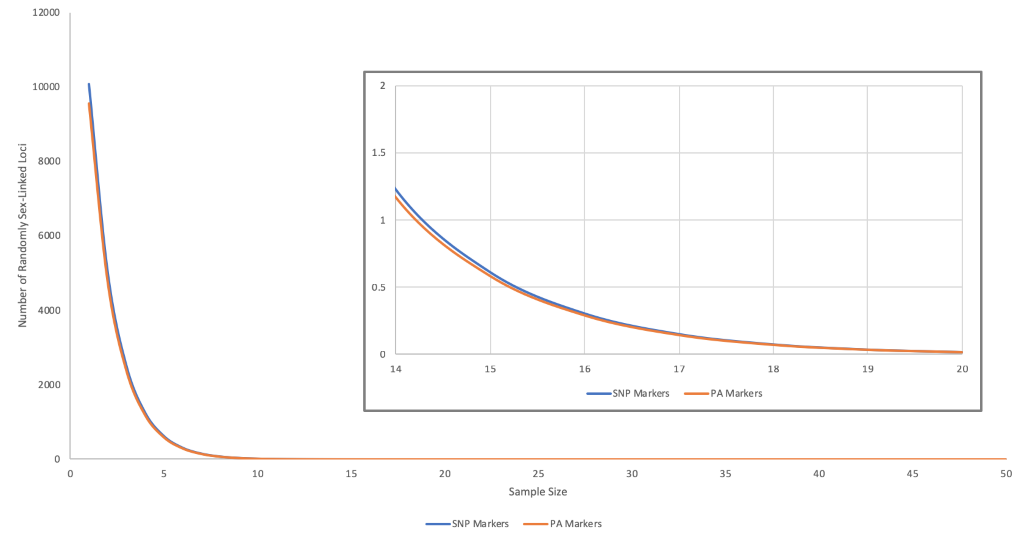


**Supplementary Figure S3.** Estimation of random sex-linked loci derived from the equation *P_i_* = 0.5*^n^*, where *P_i_* is the probability that a marker is spuriously sex-linked, and *n* is the sample size. From this, *P_i_* is multiplied by the number of quality SNP markers (20,111) and PA markers (19,121) in this study, and the number of expected loci to be randomly sex-linked for a range of sample sizes is shown ^9^.
